# The interacting physiology of COVID‐19 and the renin‐angiotensin‐aldosterone system: Key agents for treatment

**DOI:** 10.1002/prp2.917

**Published:** 2022-02-01

**Authors:** Eugenie R. Lumbers, Richard Head, Gary R. Smith, Sarah J. Delforce, Bevyn Jarrott, Jennifer H. Martin, Kirsty G. Pringle

**Affiliations:** ^1^ School of Biomedical Sciences & Pharmacy University of Newcastle Newcastle New South Wales Australia; ^2^ Hunter Medical Research Institute New Lambton Heights New South Wales Australia; ^3^ University of South Australia Adelaide South Australia Australia; ^4^ VP System Practice International Society for the System Sciences Pontypool UK; ^5^ Florey Institute of Neuroscience & Mental Health University of Melbourne Parkville Victoria Australia; ^6^ Centre for Drug Repurposing and Medicines Research Clinical Pharmacology University of Newcastle Newcastle New South Wales Australia

**Keywords:** ACE2, COVID‐19, inflammation, renin‐angiotensin‐aldosterone system, therapies

## Abstract

SARS‐CoV‐2 interacting with its receptor, angiotensin‐converting enzyme 2 (ACE2), turns the host response to viral infection into a dysregulated uncontrolled inflammatory response. This is because ACE2 limits the production of the peptide angiotensin II (Ang II) and SARS‐CoV‐2, through the destruction of ACE2, allows the uncontrolled production of Ang II. Recovery from trauma requires activation of both a tissue response to injury and activation of a whole‐body response to maintain tissue perfusion. Tissue and circulating renin‐angiotensin systems (RASs) play an essential role in the host response to infection and injury because of the actions of Ang II, mediated via its AT_1_ receptor. Both tissue and circulating arms of the renin angiotensin aldosterone system's (RAAS) response to injury need to be regulated. The effects of Ang II and the steroid hormone, aldosterone, on fluid and electrolyte homeostasis and on the circulation are controlled by elaborate feedback networks that respond to alterations in the composition and volume of fluids within the circulatory system. The role of Ang II in the tissue response to injury is however, controlled mainly by its metabolism and conversion to Ang‐(1‐7) by the enzyme ACE2. Ang‐(1‐7) has effects that are contrary to Ang II‐AT_1_R mediated effects. Thus, destruction of ACE2 by SARS‐CoV‐2 results in loss of control of the pro‐inflammatory actions of Ang II and tissue destruction. Therefore, it is the response of the host to SARS‐CoV‐2 that is responsible for the pathogenesis of COVID‐19.

## Graphical abstract

When SARS‐CoV‐2 enters cells by binding to ACE2; it stimulates local Ang II production. This recruits innate defence mechanisms. It also destroys ACE2 and so leaves Ang II‐AT1R induced inflammation unchecked.

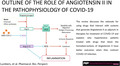

**Abbreviations**
ACE2: angiotensin‐converting enzyme 2
ACEIs: angiotensin converting enzyme inhibitors
AGT: angiotensinogen
Ang II: angiotensin II
APA: aminopeptidase A
APN: Aminopeptidase N
ARB: angiotensin receptor blocker
COX‐2: cyclooxygenase‐2
DC: Dendritic cell
GM‐CSF: granulocyte macrophage colony stimulating factor
GPCR: G protein‐coupled receptor
GPx: glutathione peroxidase
ICAM‐1: intercellular adhesion molecule‐1
IRAP: insulin regulated aminopeptidase
MD: macula densa
MgrD: Mas‐related G protein‐coupled receptor D
MLDAD: Mononuclear Leucocyte‐Derived Aspartate Decarboxylase
NO: nitric oxide
PKC: protein kinase C
PLP: proteolipoprotein peptide
RAAS: renin angiotensin aldosterone system
RASs: renin‐angiotensin systems
ROS: reactive oxygen species
SNS: sympathetic nervous system
SOD: superoxide dismutase
TAC: total antioxidant capacity
VCAM‐1: vascular cell adhesion molecule‐1
VEGF: vascular endothelial growth factor

## 1 INTRODUCTION

### 1.1 SARS‐CoV‐2 and the ACE2 receptor

This article describes how SARS‐CoV‐2, by interacting with its receptor, angiotensin converting enzyme 2 (ACE2), turns the host response to infection into a dysregulated uncontrolled inflammatory response. We describe the multiple pathways via which angiotensin (Ang) II is involved in the inflammatory response and, with the evidence presented in this article, make the case that blockade of the actions of Ang II should be seriously considered as a therapeutic pathway for patient treatment in COVID‐19. This conclusion is supported by a meta‐analysis of 52 studies of 101 949 patients with COVID‐19 infection which showed that patients who received drugs that block the formation or actions of Ang II had a lower risk of severe adverse reactions or mortality.^1^

Pandemic inducing coronaviruses like SARS‐CoV‐2, relatively harmless coronaviruses such as HCoV‐NL63, and lethal coronaviruses such as SARS‐CoV, all utilize ACE2 to enter cells and replicate. SARS‐CoV‐2 and SARS‐CoV are betacoronaviruses while HCoV‐NL63 is an alphacoronavirus.^2^ These seem to be the only viruses identified that cause infection in humans and bind to the ACE2 receptor. However, in an extensive study of bat SARS‐like coronaviruses, Ge et al. found that bat SL‐CoV‐WIVI also binds to hACE2.^3^

ACE2 is a highly conserved protein, it appeared early in evolution, about 550 million years ago, as it is found in chordates like *Ciona intestinalis*.^4^ All examples of human viruses with broad host ranges use highly conserved cell receptors (i.e., with more than 90% amino acid sequence homology).

**FIGURE 1:**
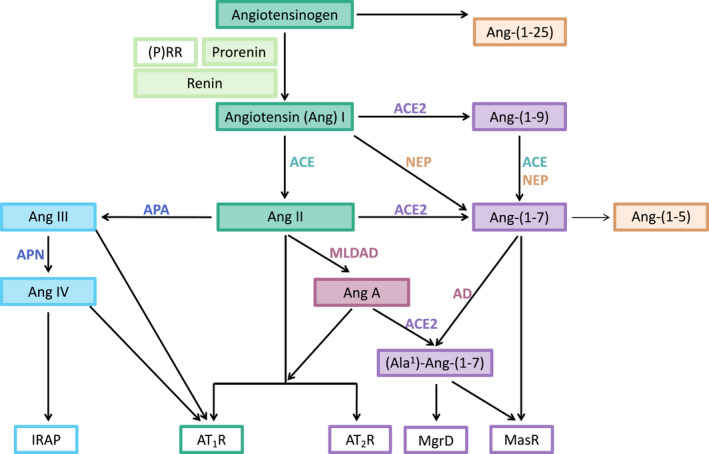
Angiotensin peptides, their synthesis, and their receptors. Green boxes indicate those pathways activating the pro‐inflammatory Angiotensin II type 1 receptor (AT_1_R). Purple boxes indicate those pathways that result in formation or actions of anti‐inflammatory Ang peptides. Active renin is the major catalytic enzyme that produces Angiotensin (Ang) I from angiotensinogen. Other proteases can produce Ang‐(1‐12) and (1‐25) from angiotensinogen. These peptides can then be catalyzed to smaller active Ang peptides. Prorenin is the inactive form of renin which can produce Ang I if bound to the prorenin receptor or if activated by proteases (see Figure 2). Angiotensin converting enzyme (ACE) converts Ang I to Ang II, the major peptide acting on the AT_1_R, which can also act on the AT_2_R. Ang II can be metabolized by aminopeptidase A (APA) to Ang III and Ang IV by Aminopeptidase N (APN). Ang IV is a ligand for insulin regulated aminopeptidase (IRAP). Ang II is the major substrate for ACE2, which converts it to Ang‐(1‐7). Ang‐(1‐7) can also be formed from Ang II via Ang A (which is produced by Mononuclear Leucocyte‐Derived Aspartate Decarboxylase (MLDAD) and ACE2, which converts it to Alamandine (Ala‐Ang‐(1‐7)). Thus, there are alternative pathways that can generate Ang‐(1‐7), see also its formation from Ang I by the interaction of ACE2 and neprilysin (NEP). Ang‐(1‐7) acts on the MasR and the AT_2_R and Ang‐(1‐7) and Ala^1^Ang‐(1‐7) can act on Mas‐related G protein‐coupled receptor D (MgrD). AD, Aspartate Decarboxylase^6^, ^7^

ACE2 is a component of the renin‐angiotensin system (RAS, Figure 1). The RAS is an ancient system with most genes appearing in the Paleozoic period, so that the key components of the RAS pathways (Figure 1) are present in amphibians.^4^ Since angiotensin (Ang) II is the major peptide formed by the RAS and its major receptors (AT _1_ R and AT _2_ R) also appeared very early, close to the appearance of ACE and ACE2,, it follows that Ang II has been biologically significant throughout evolution.^4^ It is this system that is responsible for the pathological host response, including morbidity and death, to COVID‐19 infection. To understand the pathophysiology of COVID‐19 and before potential treatments of COVID‐19 can even be considered, it is therefore necessary to understand the role(s) of this ancient system and of ACE2.

**FIGURE 2:**
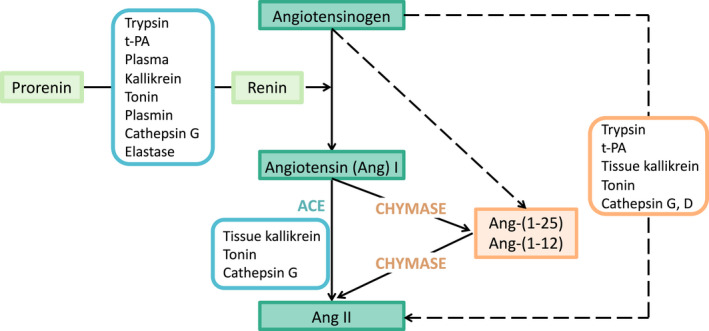
Alternate pathways that can produce Angiotensin (Ang) II. These pathways are important for cellular and tissue production of Ang II. Dotted lines indicate the pathways where intermediate peptides have not been described. t‐PA, tissue plasminogen activator. Note some of these enzymes can activate prorenin as well as forming Ang II from larger Ang peptides. Derived from Belova^8^

Primitive *Homo sapiens* first emerged about 300 000 years ago as hunter gatherers. They lived in small groups with minimal exposure to infection but maximum exposure to trauma. Trauma involves injury, damage to tissues and cells, and blood loss. To recover from trauma requires activation of both a tissue response to injury/infection and activation of a whole‐body response to maintain tissue perfusion. Tissue and circulating RASs fulfill both roles. Thus, the RAAS (renin‐angiotensin aldosterone system) can be seen as an integrated system acting at both the cellular level and as an endocrine system coordinating the whole‐body response to injury/infection.

Ang II interacting with its receptor, the Ang II type 1 receptor (AT_1_R), is the major pathway coordinating this “emergency” response system. Although the Ang II‐AT_1_R pathway is essential for survival, from the time of birth it can, if overexpressed, cause damage to both tissues and systems. This is most clearly demonstrated by the fact that many of the comorbidities suffered by *Homo sapiens* in the 21st century are due to damage that involves an overactive Ang II‐AT_1_R pathway (e.g., damaging effects of diabetes mellitus, hypertension, glomerulosclerosis, and cardiac fibrosis).

We propose that both the circulating (RAAS) and tissue renin‐angiotensin (RAS) systems are always tonically active, and that these and other Ang II forming systems activate the AT_1_R to maintain cellular and tissue integrity both by local paracrine/autocrine actions and by integration of homeostatic regulation of tissue perfusion and blood pressure. ACE2 is the major protease regulating the activity of the Ang II‐AT_1_R although there are other pathways that can also degrade Ang II to produce Ang‐(1‐7) (Figure 1).

When SARS‐CoV‐2 enters cells by binding to ACE2; it stimulates local Ang II production. This recruits innate defense mechanisms. It also destroys ACE2 and so leaves Ang II‐AT_1_R induced inflammation unchecked. It follows that if the viral load is low, the effects of SARS‐CoV‐2 infection will minimal, but in those situations in which Ang II production is tonically activated, for example, obesity, hypertension, and diabetes mellitus, where it is likely that ACE2 receptor density is increased, SARS‐CoV‐2 will infect tissues more avidly, the Ang II‐AT_1_R pathway will be activated more intensely, and the inflammatory response will be more severe.

A clear example of this that ACE2^y/−^ mice do not get SARS‐CoV spike protein induced lung inflammation whereas ACE2*^y^* ^/+^do.^9^ Thus it is not surprising that obesity also induces a more severe inflammatory response and worse outcomes in patients with COVID‐19.^10^

Some patients infected with SARS‐CoV‐2 experience a hyper‐inflammatory response known as a “cytokine storm.” It is this overwhelming release of pro‐inflammatory cytokines and, in the case of COVID‐19, suppressed levels of anti‐inflammatory cytokines that causes the severe lung and tissue damage seen in COVID‐19. Tisoncick et al. describe a cytokine storm as the outcome of a series of overlapping networks each with a degrees of redundancy and alternate pathways which can be induced by multiple infections and was first described in graft versus host disease.^11^ Mahmudour et al. claim that there are four interlinking networks responsible for the cytokine storm induced by SARS‐CoV‐2.^12^ Pivotal to this inflammation is the downregulation of ACE2 and activation of the ACE‐Ang II‐AT_1_R axis, suppression of the ACE2‐Ang‐(1‐7)‐MasR axis and activation of the complement system and [des‐Arg9]‐bradykinin systems.

This article describes the role of the RAAS in the host response to COVID‐19 infection because it is the components of this system (activation of the ACE‐Ang II‐AT_1_R axis, suppression of the ACE2‐Ang‐(1‐7)‐MasR axis) that initiate and determine the morbidity and mortality associated with this infection. However, when discussing the host response to SARS‐CoV‐2, it is important to realize that:

1. there are a number of enzymes besides renin that can form Ang II, and a number of peptides other than Ang II that are capable of activating the AT_1_R, which could play significant roles in the pathogenesis COVID‐19 infection (Figures 1 and 2);
2. there are also proteins that can mimic the action of Ang II, for example, autoantibodies that are AT_1_R agonists^13^ and the soluble prorenin receptor,^14^ that can activate AT_1_R;
3. the pathways that produce Ang peptides also interact with other peptidergic systems, for example, kallikrein‐bradykinin pathways which can have opposing actions^15^ or novel pro‐inflammatory effects which, in combination with Ang II, contribute to the cytokine storm of severe COVID‐19^16^;
4. the inflammatory response involves the migration of cells to the site of infection (partly through the effects of locally produced Ang II). As these cells contain Ang II‐producing pathways, they bring the peptide to the site of inflammation and so enhance the Ang II‐AT_1_R induced response within the tissue
5. the degree of protection offered by the ACE2 pathway in preventing severe inflammation cannot be over emphasized. ACE2 is anti‐inflammatory because it:
  - metabolizes Ang II;
  - converts Ang II to Ang‐(1‐7); and
  - Ang‐(1‐7) acts via MasR, AT_2_R, and MgrD to block pro‐inflammatory pathways mediated by Ang II and other peptides that interact with the AT_1_R (Figure 1).

Thus, destruction of ACE2 results in overexposure of cells to Ang II. Since Ang II can be produced by both extracellular and intracellular pathways this means that both blockade of ACE by angiotensin converting enzyme inhibitors (ACEIs) and Ang II‐AT_1_R receptor blocking drugs (ARBs) may not necessarily fully protect against the loss of ACE2 because they cannot enter the cell.

## 2 THE REGULATION AND ACTIONS OF THE CIRCULATING AND TISSUE RENIN‐ANGIOTENSIN SYSTEMS

### 2.1 The circulating renin‐angiotensin aldosterone system (RAAS)

**FIGURE 3:**
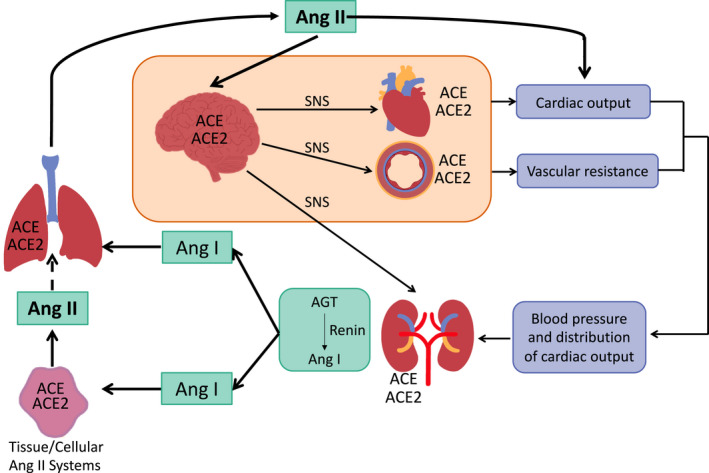
Key organs involved in the circulating renin‐angiotensin system (RAS). Renin forms angiotensin (Ang) I from angiotensinogen (AGT). Renin secreted into renal venous blood and renal lymph forms Ang I. Angiotensin converting enzyme (ACE) converts Ang I to Ang II; ACE2 converts Ang II to Ang‐(1‐7). Note that although kidney and tissues can form Ang II from Ang I, a major site for the conversion of circulating Ang I to Ang II is the lung. Ang II enters the arterial circulation and acts on the brain, activating sympathetic outflow and inhibiting parasympathetic (vagal) outflow. Thus, centrally acting Ang II affects cardiac output and peripheral vascular resistance via the sympathetic nervous system (SNS) and directly affects blood vessels and cardiac output so that blood pressure is increased. Renal secretion of renin is controlled by renal baroreceptors, the renal SNS and the macula densa. ACE2 is found in all sites where Ang II acts via AT_1_Rs. Thus SARS‐CoV‐2 can infect renal, vascular, lung, brain, and cardiac tissues (possibly by vascular endothelial infection) as well as any other tissues possessing ACE2 (gut, pancreas, testis, and ovary). Destruction of ACE2 in these tissues causes inflammation, organ dysfunction and ultimately fibrosis

Only the kidney releases both active renin and inactive renin (prorenin) into the circulation. Production of Ang II by active renin from its substrate angiotensinogen (AGT), is finely controlled by several feedback pathways that can be simple and direct, that is, mediated by Ang II interacting with its AT_1_R (blocking renin release), or elaborate and indirect. Figure 3 shows the major organs involved in the integrated control of circulating Ang II concentrations. Ang II controls aldosterone secretion from the zona glomerulosa of the adrenal gland which, along with the intrarenal RAS, is critical in maintaining/restoring blood volume.

The major complex feedbacks controlling active renin release from juxtaglomerular cells in the renal afferent arteriole can be direct or involve intermediary neural pathways such as high‐ and low‐pressure baroreceptors. They include control by renal perfusion pressure and the macula densa (MD), a sensor of intra‐renal tubular Na^+^ concentration as well as centrally mediated renal sympathetic nerve activity. Thus, the circulating RAS is controlled in response to homeostatic demand mediated by controlling renin release.

Angiotensinogen (AGT), the substrate of renin, is formed in the liver. Its production is stimulated by estrogens and by Ang II.^17^ Therefore, there is, a feed forward mechanism involved in controlling circulating AGT and a feedback system controlling renin secretion. It should be noted that although these organs have been highlighted as playing critical roles in the formation and control of the endocrine RAS they also possess paracrine/autocrine RASs which together with the endocrine RAS make them more susceptible to the adverse effects of destruction of ACE2 by SARS‐CoV‐2.

Angiotensin II, which is increased in patients with COVID‐19, stimulates aldosterone production via its actions on the AT_1_R. Aldosterone is a key effector in the RAS and is involved in sodium homeostasis. Aldosterone is however regulated by other humoral factors, for example, ACTH, sodium, and potassium concentrations. Aldosterone acts via its mineralocorticoid receptor (MR) which also has pro‐inflammatory actions.^18^ With destruction of ACE2 by SARS‐CoV‐2, Ang II levels rise leading to increased aldosterone levels. Aldosterone and Ang II acting in concert cause inflammation^19^ because like Ang II, aldosterone activates NADPH and the NF‐κB pro‐inflammatory pathway.^20^ Drugs like spironolactone and eplerenone, which block the mineralocorticoid receptor (MR), are anti‐inflammatory and anti‐fibrotic. MRs as well as Ang II‐AT_1_Rs occur in cells of the innate immune system. Aldosterone works in concert with Ang II in promoting the host response to COVID‐19, particularly because Ang II through the generation of ROS makes the MR no longer aldosterone selective, leading to generation of ATP, activation of Weibel‐Palade bodies, clotting and increased capillary permeability.^19^

### 2.2 Tissue protease (renin)‐angiotensin systems

A number of enzymes in addition to renin can produce Ang II (Figure 2) and, in tissues, there is no complex homeostatic regulation of these angiotensin producing systems such as those controlling the circulating RAS. Tissue angiotensin‐producing systems, many of which express renin, have been found in most organs, tissues, and cells of the body (e.g., heart, lung, brain, eye, gut, male and female reproductive tracts and kidneys) as well as adipose tissue.^21^

Tissues do not secrete active renin, they constitutively release prorenin. To produce Ang I from AGT, prorenin has to be activated either by proteolytic enzymes (such as trypsin, cathepsin D and plasmin) that remove its 28 amino acid pro‐segment^22^, ^23^, ^24^ or by binding of prorenin to the (pro)renin receptor ((P)RR).^25^ Prorenin binding to the (P)RR results in unfolding of the prorenin molecule so that its catalytic site is exposed and AGT can bind to it and form Ang I. (P)RR forms an integral part of membrane bound vacuolar (v)‐ATPase. Since v‐ATPases result in the extrusion of protons it is tempting to suggest that the (P)RR in conjunction with its ability to bind and unfold prorenin, is involved in converting prorenin to active renin by lowering extracellular pH and activating prorenin cleaving enzymes such as cathepsin D.^26^ Cathepsin D is increased in plasma following myocardial infarction and may contribute to Ang II production^27^ like other enzymes (Figure 2). (P)RR can be produced as an independent moiety by lysosomal enzymes and is secreted in a soluble form (s(P)RR) which increases the catalytic activity of renin on oxidized AGT^28^ but can also act as an AT_1_R agonist.^14^

For renin to produce Ang II and activate the AT_1_R, Ang I, the decapeptide produced by its action on AGT, must be converted to Ang II by the dipeptidase ACE (Figure 1). ACE is an ectoenzyme situated on the cell membrane. While ACE controls the formation of Ang II it also degrades another peptide, bradykinin, producing des‐Arg9‐bradykinin (DABK). DABK acts as an agonist on the B1 bradykinin receptor, which is activated in inflammation and upregulated when ACE2 destroyed.^29^

ACE2, a homologue of ACE is also membrane‐bound and metabolizes Ang II, forming Ang‐(1‐7) and DABK. Thus, production and hydrolysis of angiotensin and bradykinin peptides are linked because the two key proteases of the RAS, ACE, and ACE2 (Figure 1) control their production and actions.^30^

It is also important to remember that prorenin, renin and ACE are not the only enzymes capable of forming Ang II from AGT or Ang I. Chymase, trypsin, and cathepsins can also generate Ang II (Figure 2).^8^

#### 2.2.1 Chymase: A significant alternative Ang II producing pathway

Chymase can convert Ang I to Ang II and is probably the major converting enzyme in the heart.^31^ Chymase is also the major enzyme responsible for producing Ang II from Ang‐(1‐12) (Ang I‐Val^11^‐Ileu^12^).^32^, ^33^ This pathway is obviously resistant to inhibition by renin inhibitors or by ACE inhibitors. Chymase can also cleave Ang II from BigAng25 (BAng‐25), a 13 amino acid extended C‐terminus peptide of Ang‐(1‐12). BAng‐25 has been found in urine. These other Ang II producing pathways may be significant for intracrine production of Ang II. Since neither ACE inhibitors (ACEIs) nor angiotensin receptor blockers (ARBs) can target these intracellular pathways these mechanisms of production of Ang II are resistant to their actions.^34^

#### 2.2.2 Feedback mechanisms that control the production and activity of tissue angiotensin‐AT_1_R interactions

Feedback mechanisms for controlling the activity of tissue RASs are not as refined as those that exist for managing the circulating RAAS. The major enzyme degrading Ang II and producing Ang‐(1‐7) is ACE2, although there are alternate pathways that produce Ang‐(1‐7) which, by interacting with AT_2_R, MgrD, or MasR, has anti‐inflammatory actions (Figure 1). However, Ang II is the major substrate for ACE2, and its removal by ACE2 is paramount to reducing its pro‐inflammatory effects. There is evidence that ACE2 is upregulated when tissue Ang II peptides are increased, for example, in the heart, in cardiac myocytes in both dilated and hypertrophic cardiomyopathy^35^ but whether this is a simple feedback mechanism due to the Law of Mass action or involves more complex intracellular control is unknown. Another receptor known to counteract the actions of Ang II on the AT_1_R is the AT_2_R, which is also found to be more transiently but highly expressed in inflamed and healing tissues in adults.^36^

#### 2.2.3 ACE2

ACE2 controls the anti‐inflammatory arm of the RAS. It is a monocarboxypeptidase that can also produce Ang‐(1‐9) from Ang I (Figure 1). Acting in conjunction with ACE, ACE2 can therefore produce Ang‐(1‐7) via this route. However, its major substrate is Ang II and its ability to metabolize Ang II must be considered significant in terms of limiting the actions of Ang II at the AT_1_R. The role of ACE2 as a receptor for SARS‐CoV, SARS‐CoV‐2, and other coronaviruses means it plays a pivotal role in the pathogenesis of these viral infections. ACE2, like other components of the angiotensin producing pathways, is ubiquitous. It is an ectoenzyme hence SARS‐CoV‐2 can readily access it and enter cells. The nasopharynx and gastrointestinal tracts are portals for entry of the virus.

Briefly, the SARS‐CoV‐2 spike protein (S protein) is essential for binding and entry to ACE2 into the cell; it has two functional components which must be separated from each other for complete viral entry. The S1 domain binds to ACE2 via its receptor‐binding domain (RBD) which contains the receptor‐binding motif (RBM, 438‐506). The RBM binds to ACE2 and determines the affinity of SARS‐CoV‐2 for ACE2. The S2 domain contains three sequences necessary for cell entry. The spike contains a proprotein convertase (PPC) subunit at the S1/S2 boundary, which Shang et al. showed to be critical for viral entry.^37^ Furin is responsible for S1/S2 cleavage. Lysosomal cathepsins and the surface protease, TMPRSS2, also facilitate cell entry. Shang et al. showed that all three of these enzymes (furin, cathepsin and TMPRSS2) cumulatively facilitate cell entry but furin pre‐activation allows the virus to enter a variety of cells more readily, including those low in the expression of TMPRSS2 and cathepsins.^37^ Protease activation causes a structural change in the virus essential for its fusion via S2 with the cell membrane. Although SARS‐CoV‐2 RBD binds to ACE2 with a greater affinity than SARS‐CoV RBD^38^ the spike protein itself has an affinity similar to or less than SARS‐CoV. This is because the RBD of SARS‐CoV‐2 is most often hidden from the surface which enhances its ability to evade immune defense mechanisms, but it has to “stand up” to bind to ACE2. The SARS‐CoV RBD is on the other hand usually in a “standing up” position.^37^

Descending infections of the respiratory tract cause inflammation in the lung. In the gastrointestinal tract ACE2 is most highly expressed in the small intestine where it is found on enterocytes and is involved neutral amino acid transport. ACE2 expression increases with age.^39^ Within the body SARS‐CoV‐2 gains access to heart and kidneys via infection of the vascular endothelium; similar infection of the brain vascular system is due to endothelial spread of the virus and subsequent vascular damage.

The density of gut ACE2 may affect the viral load of an individual exposed to SARS‐CoV‐2 and destruction of ACE2 in the gut by SARS‐CoV‐2 could cause diarrhea and other gastrointestinal symptoms found in COVID infected patients.^40^

#### 2.2.4 Ang peptides, receptors and signalling pathways

Both the AT_1_R and AT_2_R are seven transmembrane proteins belonging to the G protein‐coupled receptor (GPCR) super family. Ang II dependent activation of AT_1_R is transient and results in receptor internalization. Forrester et al.^41^ state *Ang II activation of AT_1_R promotes signaling that is diverse, convergent, and convoluted*. As far as is currently known the AT_1_R is the only angiotensin receptor responsible for the pro‐inflammatory actions of angiotensin peptides.

#### 2.2.5 AT_1_R

Activation of the AT_1_R by Ang II causes its interaction with the heterotrimeric G proteins, including Gq/11, G12/13, and Gi. Second messenger signaling includes inositol triphosphate, diacylglycerol, arachidonic acid, and reactive oxygen species (ROS) resulting in activation of downstream effectors including phospholipases C, A and D. AT_1_R also activates various intracellular protein kinases. The responses of tissues to these pathways can differ, resulting in different effects within different tissues.

AT_1_Rs can homodimerize and form heterodimers but their physiological significance is obscure. Formation of dimers alters AT_1_R signaling; AT_1_R dimerization with AT_2_R, endothelin B receptor, or Mas may be inhibitory while dimerization with B1 and B2 adrenoceptors, Bradykinin B2, CCR2, PGFR, and P2YR6 are stimulatory.^41^

**FIGURE 4:**
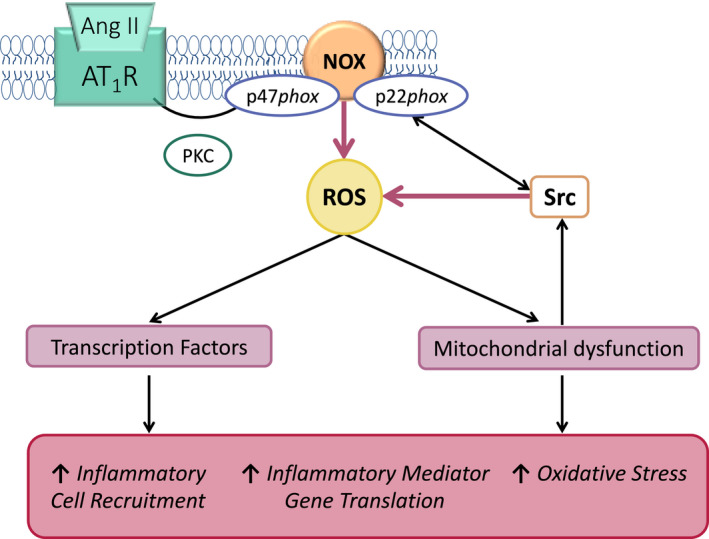
Angiotensin II‐mediated ROS activation. Ang II‐AT_1_R signaling stimulates NAD(P)H oxidase (NOX) via protein kinase C (PKC) to generate reactive oxygen species (ROS). This in turn causes mitochondrial dysfunction and activates Src kinase, further generating ROS. Together these pathways cause oxidative stress, promote translation of inflammatory genes, and recruit inflammatory cells to the tissue

A major pathway for Ang II‐AT_1_R signaling is stimulation of NADH oxidases and the production of superoxides and H_2_O_2_ (Figure 4).^42^ Griendling et al.^43^ first showed that treatment of vascular smooth muscle cells (VSMCs) with Ang II caused a 3‐fold increase in O_2_^−^ by activation of NADH and NADPH oxidases with p22 phox as the obligatory component; p22phox is the alpha subunit of cytochrome b558 and the final electron transporter from NADPH to molecular oxygen. Unlike phagocytic NAD(P)H oxidases (NOX)^44^ that generate O_2_ in a burst‐like manner extracellularly, nonphagocytic NOX enzymes are constitutively active, produce O_2_^−^ intracellularly in a slow and sustained fashion, and act as intracellular signaling molecules, influencing not only transcription factors but also other molecules involved in inflammation.^42^ Ang II stimulates the expression and catalytic activity of NOX proteins within both cytosol and mitochondrial fractions (Figure 4).^42^

Different effects occur in different cells, for example, NOX4 derived ROS contribute to fibrotic responses of mesangial cells while Ang II activation of NOX5 via Ca^++^ and calmodulin leads to growth and inflammatory responses in human vascular endothelial cells. Generation of ROS seems to underpin most of the deleterious actions of Ang II in most tissues, damage that ranges from the genome^45^ to the lung.^46^ Ang II is rapidly internalized by cells and can interact with nuclear and mitochondrial AT_1_Rs.^47^ Intracellular Ang II activates nuclear factor kappa‐light‐chain‐enhancer of activated B cells (NF‐κB), and other pro‐inflammatory molecules.^48^

#### 2.2.6 AT_2_R

Ang II acting via the AT_2_R generally has effects that oppose those mediated by its interaction with AT_1_R however this is not always the case. Like the AT_1_R, the AT_2_R is a GPCR so it has diverse downstream effects depending on cell type. Ang II‐AT_2_R interactions inhibit ERK and MAPK, and promote vascular relaxation through PKA‐dependent endothelial nitric oxide synthase (eNOS) activation and paracrine signaling through bradykinin/cyclic GMP/nitric oxide (NO) production.^49^ The AT_2_R‐interacting protein family, ATIP1‐ATIP4, promotes an anti‐inflammatory environment. ATIP1 is the mitochondrial suppressor gene 1 (MTSG1) which, when over expressed in cardiac myocytes, inhibits phenylephrine induced mitochondrial ROS production, ERK activation and hypertrophy.^50^

#### 2.2.7 MasR

The activation of Mas, a G‐protein‐coupled receptor (GPCR) is implicated in leukocyte recruitment, inflammation, and vascular relaxation.^51^ Ang‐(1‐7) acts via the MasR. The protective actions of Ang‐(1‐7) interactions with AT_2_Rs such as vasodilation are independent of the MasR.

#### 2.2.8 Mas‐related G protein‐coupled receptor D (MgrD)

MgrD is a member of the Mas related GPCR family that counteracts Ang II‐AT_1_R actions. It is activated by Ang‐(1‐7) to produce arachidonic acid and PKA‐CREB‐dependent phosphorylation (cyclic AMP response element binding protein), which contributes to vasorelaxation. NO production is also increased by the action of alamandine acting on MgrD (Figure 1). Therefore, Ang‐(1‐7) opposes the actions of Ang II‐AT_1_R through its interactions with three different receptors.

## 3 INFLAMMATION AND CELLULAR ANGIOTENSIN‐PRODUCING SYSTEMS

**FIGURE 5:**
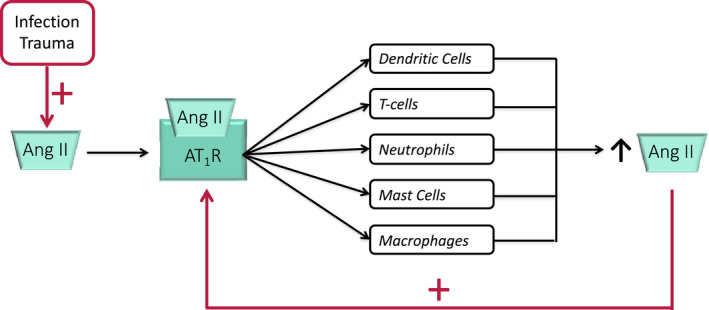
Summary of the positive interactions between the renin‐angiotensin system (RAS) and the innate immune system. Angiotensin (Ang) II has the capacity to affect the activities and properties of these immune cells. They in turn have the capacity to generate Ang II thus reinforcing the pro‐inflammatory actions of Ang II, particularly through the generation of reactive oxygen species (ROS)

The innate immune system is the first line of defense against trauma and infection. Inflammation damages tissues and organs, and repair is often associated with fibrosis. Three main pathways (NFκB, MAPK, and JAK‐STAT) play major roles in inflammation, and dysregulation of one or more of these pathways can lead to inflammation‐associated disease.^52^ What is commonly missing from publications, is the role of Ang II‐AT_1_R and aldosterone‐MR in inflammation even though they complement the generation of ROS by cells involved in the immune response, stimulate key signaling pathways involved in the inflammatory response, for example, NF‐κB and MAPK, and recruit immune cells to sites of injury/infection (Figure 5). This section deals with tissue angiotensin producing systems and their response to injury or infection.

### 3.1 Ang II‐AT_1_R activation and amplification of inflammatory pathways

Tissue (renin) angiotensin producing systems are activated locally. Signaling pathways are described above but Ang II‐AT_1_R also increases vascular permeability by promoting the secretion of vascular endothelial growth factor (VEGF)^53^, ^54^ and inducing expression of endothelial adhesive molecules including selectins (P‐ and L‐Selectin), vascular cell adhesion molecule‐1 (VCAM‐1), intercellular adhesion molecule‐1 (ICAM‐1) and their ligands, the integrins.^55^ Ang II also promotes endothelial dysfunction through cyclooxygenase‐2 (COX‐2) activation, which generates vasoactive prostaglandins and ROS.^56^, ^57^

Ang II‐AT_1_R production of ROS amplifies the inflammatory actions of the innate immune system (Figure 5). Aldosterone acts in synergy with Ang II as many cells of the innate immune system also possess MRs (see above).

### 3.2 Dendritic cells

Dendritic cells (DCs) are formed from bone marrow progenitors which home to tissues in which they reside until stimulated by local infection or inflammation. Immature DCs actively capture and process antigens and enter a terminal differentiation phase. At this time, they leave their tissues and migrate to the T cell‐dependent areas of secondary lymphoid organs (i.e., spleen and lymph nodes) increasing their ability to stimulate resting T cells and so initiating immune responses.^58^

The differentiation of DCs from precursor circulating monocytes was studied by Nahmod et al.^58^ in the presence of the Ang II‐AT_1_R antagonist, losartan. Losartan reduced expression of CD1a and reduced the ability of monocytes to endocytose material; they also appeared to have a more macrophage‐like phenotype. DC activity was potentiated if monocytes were incubated with Ang II. It was also enhanced by incubation with the AT_2_R antagonist, PD 123319. The authors also saw similar effects in mouse DCs isolated from bone marrow and cultured with granulocyte macrophage colony stimulating factor (GM‐CSF) and losartan, that is, there were low levels of CD11c, CD40, and 1a, reduced endocytic activity and reduced ability to induce antibodies. An opposite effect was seen when they were incubated with Ang II. Thus, Ang II‐AT_1_R’s interactions with DCs suggests that it regulates one of the earliest stages of the innate immune response.

### 3.3 T cells

T cells are lymphocytes, activated by exposure to a cell containing a foreign antigen. The RAS is intricately involved in T cell biology and the role of T cells in inflammation. T cells bind to cells that carry a foreign antigen; specifically, peptide fragments carried by MHC II cell surface proteins. Antigen presentation to immature CD8^+^ T cells causes maturation of cells into cytotoxic T cells that contain perforin which binds to the antigen presenting cells. Perforin forms a channel that provides a pathway for granzyme B and serine proteases like trypsin and chymotrypsin which, together with FAS ligand, destroy the antigen and induce apoptosis of infected cells.

Helper T cells (CD4^+^) can be subclassified into two main groups. Th1 cells ‘help’ macrophages by promoting their killing efficiency and stimulating proliferation of CD8^+^ T cells. Th2 cells are important in the humoral immune system, stimulating B‐cell proliferation, inducing B‐cell antibody class switching and increasing IgG, IgM, and IgA neutralizing antibodies. Significantly, T cells contain a complete RAS.^59^

Like Hoch et al.,^59^ Jurewicz et al.^60^ showed that T cells and natural killer (NK) cells are fully equipped with RAS components, can generate Ang II from AGT and Ang I and are therefore, capable of producing and delivering Ang II to sites of inflammation. They conclude that *because their chemotaxis is enhanced by Ang II, this creates a potential amplification system*. T cells express renin, AGT and ACE mRNA, and produce physiological amounts of Ang II in culture. Their AT_1_Rs are intracellular, and endogenously produced Ang II increases T cell activation, expression of the tissue homing marker, C‐C chemokine receptor type‐5 (CCR5) and production of tumor necrosis factor (TNF)‐α. This process is dependent on Ang II stimulation of NOX. Together with the intracellular actions of other components of T cells, TNF‐α (among other actions) decreases the viability of antigen presenting cells.^61^

Jurewicz et al.^60^ showed that Ang II also stimulates T‐cell proliferation. T cells populations that are depleted of NK cells do not proliferate in response to Ang II. In contrast to the mutually antagonist actions of AT_1_R and AT_2_R in maturation of DCs (see above), the interaction of Ang II with both AT_1_R and AT_2_Rs are involved in this Ang II response by T‐cells.

So profound is the role of Ang II in the immune response to inflammation that it can induce autoimmune‐induced inflammation. Autoimmune‐induced encephalomyelitis (EAE) was induced in mice with an encephalitogenic proteolipoprotein peptide (PLP).^62^ Immunization with PLP stimulated Ang II formation in CD4^+^ cells and CD11b monocytes and increased serum Ang II levels. AT_1_Rs were upregulated in lymph node cells, Ang II binding was increased in PLP‐activated CD4^+^ T cells and CD11b^+^ monocytes and in the spinal cord tissue. Interestingly, the same authors found that AT_1_Rs and other components of the RAAS were upregulated in plaques from patients with multiple sclerosis.^62^

Furthermore, in EAE mice, blocking Ang II production or activity by ACEIs or ARBs suppressed autoreactive Th1 and Th17 cells and promoted antigen‐specific CD4^+^FoxP3^+^ regulatory T cells (Treg). This inhibited the NF‐κB1 transcription factor complex induced by Ang II and activated an alternative pathway, NF‐κB2. Treatment with lisinopril, an ACEI, generated sufficient potent CD4^+^Fox3^+^ T cells to reduce paralytic EAE in mice.^62^ In another study of acquired autoimmune encephalomyelitis in mice, Stegbauer et al.^63^ showed that the RAS was involved in the inflammatory response and clinical syndrome associated with induction of EAE. They showed that the renin inhibitor, aliskiren, and the ACEI, captopril, attenuated the inflammatory response. Blockade of the AT_1_R by losartan targetted antigen presenting cells (APCs, five subsets) and blocked chemokine induced APC migration. CCL2 and CXCL10 were significantly reduced in the spleen and CCL2, CCL3, and CXCL10 were reduced in the spinal cord. Losartan also blocked the migration stimulating effects of CCL2 in macrophages. Macrophage production of CCL2 and CCL3 was reduced by prophylactic AT_1_R blockade. The authors noted that AT_1_R blockade affected CD11b^+^ and CD11c^+^ cells which would decrease macrophages in the inflamed CNS and might limit the numbers of DCs necessary to activate T cells and initiate EAE.^63^

### 3.4 Neutrophils

Intraperitoneal injection of Ang II into rats caused a rapid accumulation of neutrophils (within 4 h) by increasing levels of cytokine‐inducible neutrophil chemoattract/keratinocyte derived chemokine (CINC/KC) and macrophage inflammatory protein (MIP‐2). A CINC/KC antibody and a CXCR2 receptor antagonist abolished the effects of Ang II on neutrophil function. In human umbilical vein endothelial cells (HUVECs), Ang II releases IL‐8 by interacting with the AT_1_R.^64^ IL‐8 plays a critical role in neutrophil recruitment.^65^

Neutrophils have a surface bound cathepsin G, which is upregulated by signals associated with inflammation and infection.^66^ At this site it can convert both AGT and Ang I to Ang II, thus increasing the local production of Ang II and modulating inflammation.^67^ These authors concluded that *membrane‐bound cathepsin G expressed on neutrophils is an inducible and mobile angiotensin II*‐*generating system that may exert potent local vasoactive and chemoattractant properties at sites of inflammation*.

Interestingly, ACE contained within neutrophils increases their bactericidal activity independent of Ang II/AT_1_R pathway.^68^

### 3.5 Mast cells

Mast cells derive from CD34^+^ multi‐potent bone marrow progenitor cells. They circulate in the blood as basophils and enter mucosal surfaces and connective tissue compartments of multiple organs. They are modulators and mediators of innate immunity, inflammation, and fibrosis. Mast cells contain renin and stimulation of mast cell degranulation results in Ang I formation.^69^ Mast cells also contain chymase, the major Ang II forming enzyme in the human heart.^70^

### 3.6 Controlling Ang ‐AT_1_R activity

Because there are multiple pathways and cellular origins for the production of Ang II, any tissue subjected to inflammation is likely to be heavily infiltrated with Ang II, thus exacerbating Ang II‐AT_1_R inflammatory pathways. Although this can be offset to some extent by the anti‐inflammatory actions of Ang II mediated by its AT_2_R, this is limited. Thus ACE2 is the major regulator levels of tissues and cellular Ang II.

We postulate that inflammation, whether caused by infection, stretch or foreign antigens, releases Ang II, which recruits and interacts with pro‐inflammatory cells (described above). These cells bring Ang II forming pathways to the infected site. Since the severe host response to COVID‐19 is an overexpression of the inflammatory response to infection, inhibitors of Ang II‐AT_1_R interactions could be of therapeutic value (see below).

### 3.7 Ang II‐AT_1_R versus ACE2 and Ang‐(1‐7)

ACE2 produces Ang‐(1‐7) from Ang II (Figure 1). Ang II is its major substrate. Therefore ACE2, both by removal of Ang II and the anti‐inflammatory action of its proteolytic activity, namely Ang‐(1‐7), which acts via AT_2_R, Mas, and MgrD (see above), is the most powerful regulatory mechansim for controlling Ang II and its AT_1_R‐mediated effects.

ACE2 expression is increased in tissues like the heart when there is evidence of chronic disease.^71^ It is possible that this represents feedback regulation of Ang II’s local inflammatory actions. Therefore in tissues in which there is already chronic inflammation and increased local Ang II production, for example, in obesity,^10^ in the bronchial epithelium of overweight patients with chronic obstructive pulmonary disease (COPD^72^), and heart,^71^ ACE2 levels are increased. Since ACE2 is the portal via which SARS‐CoV‐2 enters cells to replicate, the more ACE2, the greater the viral load. Furthermore, once the spike protein of SARS‐CoV‐2 binds to ACE2, ACE2 is removed from the cell surface. This results in a significant loss of protection for tissues against inflammation.

Destruction of ACE2 by its interaction with SARS‐CoV‐2 exposes infected tissues and organs to unregulated severe inflammation and this is one reason why this virus has significant mortality and morbidity.

### 3.8 ACE2 induced cellular protection against the actions of Ang II/AT1R

The significance of the role of ACE2 in protecting against the pro‐inflammatory actions of Ang II has been clearly demonstrated (see below). These experiments show that ACE2 protects the organism from Ang II and highlights pathways and effects of Ang II, unmasked by loss of ACE2. As ACE2 is reduced with the entry of SARS‐CoV‐2 into cells, Ang II activity, including inflammation, is expected to be amplified in COVID‐19.

In an outstanding experiment, Oudit et al.^73^ showed that there were two critical aspects to Ang II‐induced inflammation: (1) The ability of Ang II to induce ROS; and (2) limitation of this action by ACE2. Briefly, in the ACE2^−/y^ mouse, a pathological cardiomyopathy developed due to increased ROS, causing a 4‐fold increase in neutrophilic infiltration, increased infiltration of inflammatory cytokines, IL‐1β, IL‐6, and monocytic chemoattractant protein (MCP), activation of MAP kinase and increased collagenase activity.^73^ All these features of ACE2^−/y^ cardiomyopathy were blocked by the angiotensin receptor blocker (ARB), irbesartan. Therefore, this chronic inflammation must have been due to Ang II (or an Ang peptide) interacting with the AT_1_R and triggering its downstream pathways. This effect was not seen in ACE2^−/y^P110γ^/−/−^ mice. The key activator of NOX that produces ROS is the catalytic subunit of PI2Kγ namely p110γ. Thus, ACE2 deficiency via increased Ang II leads to unchecked production of ROS.

The study carried out in ACE2^−/y^ mice^73^ shows that even in non‐inflamed/non‐infected tissues, endogenous tissue Ang II (or Ang peptides) cause inflammation and cardiac dysfunction when not inhibited by ACE2. Therefore, ACE2 is carrying out a physiological role in preventing overt inflammation and organ damage caused by excess Ang II and lack of Ang‐(1‐7). These authors also showed that that variable expression of NADH affected the severity of inflammation induced in ACE2^−/y^ mice, that is, the genetic background of mice influenced the effects of ACE2^−/y^ genetic deletions. Extrapolating these findings to humans suggests that genetic as well as environmental factors such as obesity and other comorbidities affect the host response to the destruction of ACE2 by SARS‐CoV‐2. This is borne out by evidence of more severe morbidity and mortality in the obese,^10^ even taking into account the difficulties in the care of ICU patients.

Rabelo et al.^74^ also studied ACE2^−/y^ mice. These mice had higher mean arterial pressures, deficiencies in antioxidants such as superoxide dismutase (SOD) and catalase although SOD2 was not decreased, and glutathione peroxidase (GPx) was in fact increased. There was also evidence of increased oxidative damage, for example, thiobarbituric acid reactive substances (TBARS). MasR knockout mice have a similar phenotype, that is, hypertension, endothelial dysfunction and an imbalance between NO and reactive oxygen species.^75^

### 3.9 The RAS, the lung, and ACE2

The role of the RAS in pulmonary vascular endothelium is extremely significant because the entire capillary network of the lung contains ACE whereas only about 20% of the capillary network in other organs express ACE.^76^ This explains the well‐known fact that the lung is the major site for the conversion of Ang I to Ang II. Thus, the lung must be rigorously protected from the pro‐inflammatory actions of Ang II (Figure 2). ACE2 is present in Clara cells and in alveolar type II cells and it is damage to these cells that is a feature of acute respiratory distress syndrome (ARDS),^77^ which is seen in many severe COVID‐19 cases.

LPS‐induced ARDS in rats was associated with increased pulmonary renin expression and activity, increased Ang II levels, increased expression of ACE and reduced expression of ACE2.^78^ These effects were reversed to some extent by treatment with Vitamin D prior to the LPS challenge. In mice, genetic knockouts of *ACE2* caused much more severe ARDS (induced by aspiration, endotoxin, or peritoneal sepsis). These animals were rescued by administration of an AT_1_R antagonist.^79^

In patients who developed ARDS compared with other patients in respiratory failure,^80^ the D/D deletion of the *ACE* gene (which is associated with increased expression of ACE) was more prevalent. This reflects the findings reported in mice^73^ showing that the genetic background influenced the severity of inflammation induced by the ACE2 knockout. Thus, it is not surprising that the SARS‐CoV spike protein, which also uses ACE2 as its receptor, exacerbated the effects of aspiration‐induced lung damage and was prevented by AT_1_R blockade.^9^

Thus, the role of Ang II in acute lung injury (ALI) is well documented as is the beneficial effect of ACE2. This has led to the suggestion that recombinant human (rh)ACE2 could be a useful therapy for ALI^81^ and potentially for SARS‐CoV‐2.

Zoufaly et al.^82^ described the case of a woman who became seriously ill with COVID‐19 requiring ventilation. She had the following clinical features of severe COVID‐19: ‘ground‐glass’ appearance of the lungs, leukopenia, thrombocytopenia, profound lymphopenia and elevated serum LDH (636 U/L), C‐reactive protein (CRP; 103 mg/L) D‐Dimer (1.3 mg/L), and ferritin (880 µg/L). Nine days after the onset of symptoms, she was treated with intravenous (rh) soluble ACE2 (sACE2) for 7 days. She developed secondary bacterial infections, but her pneumonia resolved, and she was discharged on day 57. What is interesting about this case is that the RAS was profiled before, during and after treatment. Ang II concentrations fell with (rh) sACE2 treatment while Ang‐(1‐7) concentrations rose as did Ang‐(1‐9) and their main downstream metabolite Ang‐(1‐5). As Ang II levels fell so did IL‐6, IL‐8, soluble advanced glycosylation end‐product specific receptor (a marker associated with higher mortality in ARDS), and the inflammatory marker ferritin. CRP and angiopoietin‐2 (a marker of endothelial injury) initially increased then decreased, perhaps because they were affected by concurrent bacterial infection. This paper shows that loss of ACE2 and accumulation of Ang II in the lungs was associated with pulmonary inflammation and the clinical characteristics of SARS‐CoV‐2 infected lungs. It also shows that (rh)sACE2 is catalytically active and the suppression of Ang II by its activity was accompanied by the reduction in those cytokines associated with the COVID‐related cytokine storm.^82^

What about activators of ACE2, that is, ACE2 agonists? Do they affect the outcome of ALI and could they be used in the treatment of SARS‐CoV‐2? Fang et al.^46^ induced ALI in mice by a 72 h period of hyperoxia and described the effects of an ACE2 agonist (60 mg/kg, DIZE) and an ACE2 antagonist (MN‐4760) on this acute inflammatory response. Hyperoxia caused significant lung injury, with inflammation and oxidative stress. That is, the wet:dry ratio of the lung increased, tissue permeability increased, as did protein levels in BALF (bronchoalveolar lung fluid). ACE2 levels were depressed and the Ang II/Ang‐(1‐7) ratio was increased. It could be argued therefore that hyperoxia‐induced elevation of Ang II levels caused ALI. The ACE2 antagonist (MLN‐4760/MLN‐4476) exacerbated all the inflammatory effects induced by hyperoxia. Conversely, DIZE prevented this tissue and lung damage, reversed the hyperoxia‐induced decrease in lung ACE2 and increased the Ang II/Ang‐(1‐7) ratio.

Fang et al.^46^ also showed that hyperoxia activated NF‐κB pathways (increases in phospho‐p65/p65 and decreased IK‐κb) and increased the production of pro‐inflammatory cytokines (TNF‐α, IL8, IL‐1β) in the lung. These changes were partially reversed by DIZE. The decrease in total antioxidant capacity (TAC) caused by hyperoxia was also reversed by DIZE while the elevated levels of products of oxidative stress such as malondialdehyde (MDA), TBARS, 8‐OH‐2′‐deoxyguanosine (8‐OHdG) and protein carbonyl induced by hyperoxia were decreased by DIZE treatment. Hyperoxia also decreased nuclear factor‐erythroid factor 2‐related factor 2 (Nrf2), NAD(P)H dehydrogenase [quinone] 1 (NQ01), and the heme oxygenase (HO)‐1 pathway, which activates a serine leucocyte protease inhibitor (SLPI), inhibits expression of TMPRSS2, and regulates the expression of antioxidant enzymes (e.g., SOD, catalase).^83^ DIZE reversed this effect. Therefore, ACE2 inhibits NF‐κB and enhances Nrf2, so it has a powerful anti‐inflammatory and antioxidant effects.

## 4 REPURPOSING DRUGS TO TREAT COVID‐19

**FIGURE 6:**
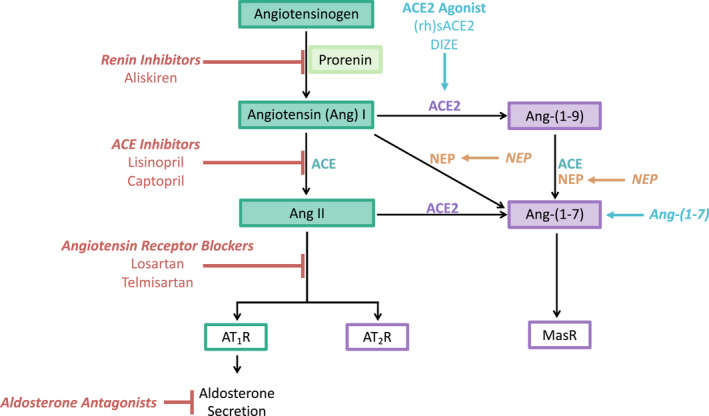
Drugs to treat COVID‐19. Repurposing existing drugs that target the RAS and would reduce the levels of Ang II or its actions on AT_1_R may be of benefit for patients with COVID‐19. These include renin inhibitors, ACE inhibitors and angiotensin receptor blockers. Drugs that block the secretion of aldosterone may also be of use. Alternatively, stimulation of the opposing RAS pathway, for example using (rh)ACE2, ACE2 agonists, or Ang‐(1‐7) may also be beneficial

This article has shown that the basis of the inflammatory response to infection with SARS‐CoV‐2 is an excess of Ang II produced both by local production in infected cells and by the destruction of ACE2, the enzyme controlling tissue concentrations of Ang II. It should be noted that there are other peptides and proteins that can interact with the AT_1_R (Figure 1), there are several proteases that generate Ang II (Figure 2) and a strong contingent of inflammatory cells recruited to sites of infection that contain Ang II producing systems. Therefore, the major therapeutic targets for treating COVID‐19 should target this imbalance between pro‐ and anti‐inflammatory RAS pathways. There are several drugs that do restore this balance, most of which are used in the treatment of cardiovascular disease, chronic renal disease and diabetes mellitus. These can be repurposed for use in treating COVID‐19 as described below (Figure 6).

### 4.1 Renin inhibitors

The renin inhibitor, aliskiren, inhibits the ability of renin to form Ang I from AGT. Guo et al. found that aliskiren was safe for treating hypertension in COVID affected patients and recommend further clinical evaluation.^84^

Aliskiren is among a number of protease inhibitors^85^ that biologically ought to be able to inhibit SARS‐CoV‐2 viral entry by blocking its major protease (M^Pro^).^86^ In fact, Aly et al. (2020) suggest it is one of the most effective of these inhibitors.^87^ M^pro^ is one of the crucial viral enzymes as it plays a key role in replication and transcription. Inhibition of M^pro^ and subsequently, the non‐structural proteins (Nsps) arrest the process of viral assembly in the SARS‐CoV‐2 replication cycle. The advantage of aliskiren therefore is that it would both block the formation of Ang II and inhibit viral entry.

### 4.2 ACE inhibitors

ACE inhibitors such as lisinopril and captopril block the formation of Ang II from Ang I. They are widely used and have been shown to reduce mortality and morbidity in COVID‐19 patients. It could be argued that ACEIs would be less effective than ARBs because although they prevent the formation of Ang II, they would also reduce Ang‐(1‐7) synthesis (see Figure 1). However, ACEIs elevate concentrations of bradykinin, which have vasodilatory effects on cardiovascular tissues which could be beneficial in the morbidity associated with COVID‐19. Studies have shown that treatment with ACEIs or ARBs is associated with a similar reduction in morbidity and mortality from COVID‐19.

Most studies that look at the impact of ACEIs in the treatment of hypertensive patients with COVID‐19 have not discriminated between ARBs and ACEIs but are associated with the significant reduction in all‐cause mortality (OR = 0.5, 95%, CI: 0.34, 0.72).^88^

Hippisley‐Cox^89^ recently reported a study of over 8 000 000 people, of whom 19 486 had COVID‐19 and 1286 received ICU care, 7.8% were treated with an ACEI and 3.7% with an ARB. ACEIs were associated with a significant reduction in COVID‐19 hospital admission (adjusted HR = 0.71, 95% CI: 0.67–0.74) and no increased risk of ICU care (after adjustment for confounders, in particular ethnicity, socioeconomic status, and gender). Adjusted ORs for ARBs were 0.63 (95% CI: 0.59–0.67) with no increase in ICU care.

Several studies have shown that ARBs and ACEIs, while safe for use in hypertensive patients with COVID‐19, do not offer any significant benefit^90^, ^91^ but these were studies directed at examining drug safety because of spurious opinions offered in the medical literature in early 2020. The outcome of prospective trials specifically designed to determine the therapeutic benefits of ACEIs and ARBs are needed.

### 4.3 ARBS

ARBs block the AT_1_R; therefore, they block the actions of Ang II produced by any pathway as well as other Ang peptides that interact with this receptor (Figure 1). Experimental use of ARBs has provided much of the information concerning the pro‐inflammatory actions of Ang II described in this article.

Duarte et al.^92^ have recently described the outcomes of a clinical trial of an ARB added to standard care of COVID‐19 patients (NCT04355936). This was a parallel‐group, randomized and two‐arm trial of the effects of telmisartan (80 mg). Eighty patients received standard care and 78 received 80 mg telmisartan. Telmisartan‐treated patients had a more abrupt and profound fall in inflammatory markers (CRP), a lower median time to discharge and fewer deaths (control 22.54%, 16/71; telmisartan 4.29%, 3/70; *p* = 0.0023). This is a dramatic effect of telmisartan and there could be other contributing factors as telmisartan is known to have off‐target effects as a peroxisome proliferator‐activated receptor (PPAR)‐γ agonist, which might also suppress the clinical syndrome of COVID‐19.

It is also feasible that blockade of the RAS by an ARB may have greater efficacy in the treatment of COVID‐19 because only the AT_1_R is blocked, therefore any Ang II formed could have anti‐inflammatory effects via its interaction with AT_2_R or via conversion by residual ACE2 to Ang‐(1‐7) which acts via AT_2_R, MasR, and MgrD (Figure 1).

### 4.4 Aldosterone antagonists

Since Ang II stimulates aldosterone secretion and there is a synergy in their pro‐inflammatory actions there could be a case for using drugs like spironolactone and epleronone that block the MR in treatment of COVID‐19. Abolishing the unique effects of MR generated by formation ATP on purinergic nerves with consequent activation of clotting and increased capillary permeability^19^ could provide additional therapeutic benefits.

## 5 ENHANCING THE ACE2‐ANG‐(1‐7) PATHWAY

Recombinant human sACE2 can “mop up” the virus in the cardiovascular system and is enzymatically active so it can increase the formation of Ang‐(1‐7) from excess Ang II. In 2020, Monteil et al.^93^ showed that (rh)sACE2 reduced SARS‐CoV‐2 infection in human organoids and later showed in vitro, that the combination of ACE2 with the anti‐viral medication, remedesvir, improved the anti‐viral activity and reduced toxicity.^94^

ACE2 has also been used to treat COVID‐19; its effects are described above.^82^ This appears to be the only cited report of clinical use of sACE2 in human COVID‐19. The anti‐inflammatory effects of ACE2 have been described (see above). (rh)sACE2 has also passed through phase I and II clinical trials (NCT00886353, NCT01597635)^95^, ^96^ for ARDS and it has now received regulatory approval in the USA for continued study. Several studies are now trialing its use for COVID‐19 (NCT04335136, NCT05065645, and NCT04924660). One of these studies is now complete (NCT04335136), however, data were inconclusive.

Alternatively, Ang (1‐7) could be produced from Angiotensin I (see Figure 1) when ACE2 has been destroyed by SARS‐CoV‐2.^97^ This is the mechanism of action of the metalloprotease neprilysin which, when combined with an ACE inhibitor, is used clinically in the treatment of cardiovascular disease.

## 6 ACTIVATORS OF ACE2

### 6.1 Diminazene aceturate (DIZE)

The ACE2 agonist, DIZE, has been shown to reverse ALI, by preventing inflammation, ROS generation, NFκ‐B activation, and Nrf2 inhibition (see above^46^). Qaradakhi et al.^98^ have suggested that DIZE, through ACE2 activation, could be used to treat a wide range of disease including myocardial infarction, hypertension, and arteriosclerosis. However, Haber et al.^99^ showed that DIZE and another activator, XNT, had effects on blood pressure that must have been independent of ACE2 activation as they occurred in ACE2 knockout mice. These did not have any effects on Ang II levels and renal ACE2 activity.

### 6.2 Ang‐(1‐7) as an adjunct treatment for COVID‐19

There is a clinical trial (NCT04633772) studying the efficacy of intravenous Ang‐1‐7 for the treatment of COVID‐19. The primary endpoint is the number of supplemental oxygen‐free days by day 28. The trial will be completed in late 2021. However, it may not be as efficacious as rhsACE2 (see above) which prevents inflammation via two pathways.

## 7 SUMMARY

Ang II plays a major role in inflammation. The receptor for SARS‐COV‐2, ACE2, is intimately involved in regulating Ang II – AT_1_R interactions and inhibits the pro‐inflammatory actions of Ang II. Therefore, the destruction of ACE2 by SARS‐CoV‐2 causes dysregulation of the host response to the virus. Drugs that block the formation of Ang II or prevent its interactions with the AT_1_R are therefore useful adjuncts in the management of patients with COVID‐19. There are several trials suggesting that ACEIs and ARBs decrease mortality and morbidity from COVID‐19. Renin inhibitors, which have the same effect as ACEIs and ARBs, are also safe for use in COVID‐19 and may provide an additional therapeutic benefit by blocking viral entry into cells. It would seem however that the most efficacious treatment of patients with COVID‐19 is likely to be rhsACE2.

## 8 NOMENCLATURE OF TARGETS AND LIGANDS

Key protein targets and ligands in this article are hyperlinked to corresponding entries in http://www.guidetopharmacology.org, the common portal for data from the IUPHAR/BPS Guide to PHARMACOLOGY,^100^ and are permanently archived in the Concise Guide to PHARMACOLOGY 2019/20.^101^, ^102^

## DISCLOSURE

The authors have no conflicts of interest to declare.

## AUTHOR CONTRIBUTIONS

Eugenie R. Lumbers drafted the manuscript. All authors made substantial contributions to the conception and design of the manuscript, revised it critically for intellectual context and gave final approval of the final version.

## Data Availability

Not applicable.
